# Possible Effects of Topical Rho-Kinase Inhibitor on Schlemm’s Canal Morphology Parameters

**DOI:** 10.3390/biomedicines14020470

**Published:** 2026-02-20

**Authors:** Aysha Siddika Mukta, Aika Tsutsui, Teruhiko Hamanaka, Sachiko Kaidzu, Kanae Kobayashi, Nobuo Ishida, Masaki Tanito

**Affiliations:** 1 Department of Ophthalmology, Shimane University Faculty of Medicine, Izumo 693-8501, Japan; m259401@med.shimane-u.ac.jp (A.S.M.); kecha@med.shimane-u.ac.jp (S.K.);; 2 Department of Ophthalmology, Japanese Red Cross Hospital Medical Center, Shibuya, Tokyo 150-8935, Japan; 3 Department of Ophthalmology, Ishida Eye Clinic, Joetsu 943-0832, Japan

**Keywords:** primary open-angle glaucoma, Rho-associated protein kinase inhibitor, ripasudil, Schlemm’s canal morphology, trabecular meshwork, aqueous humor outflow

## Abstract

**Background**: To evaluate the effects of preoperative topical ripasudil, a Rho-associated protein kinase (ROCK) inhibitor, on Schlemm’s canal (SC) morphology in patients with primary open-angle glaucoma (POAG). **Methods**: This study included 95 SC specimens obtained during trabeculectomy from 95 patients with POAG. Based on preoperative treatment, patients were divided into two groups: ripasudil (−) group (*n* = 68) receiving four topical medications [FP receptor agonist, β-blocker, carbonic anhydrase inhibitor (CAI), and α_2_ agonist], and ripasudil (+) group (*n* = 27) receiving the same four medications plus ripasudil. SC morphology parameters were assessed in thrombomodulin (TBM)-stained sections, including length parameters [TBM-positive/negative and opened/closed SC lengths] and area parameters [TBM-positive/negative and opened SC areas]. Between-group comparisons were performed using unpaired *t*-tests, and multiple regression analysis was conducted to adjust for age, gender, preoperative intraocular pressure (IOP), and oral CAI use. **Results**: The ripasudil (+) group had significantly longer total SC length (TSC: 302.5 µm) than the ripasudil (−) group (273.0 µm, *p* = 0.023). Among area parameters, the ripasudil (+) group showed significantly larger opened SC area (OSC-A: 2689 µm^2^ vs. 1881 µm^2^, *p* = 0.008) and TBM-negative opened SC area (NOSC-A: 716 µm^2^ vs. 305 µm^2^, *p* = 0.001), whereas TBM-positive opened SC area (POSC-A) was not significantly different between groups (2001 µm^2^ vs. 1575 µm^2^, *p* = 0.096). After multivariate adjustment, ripasudil use remained significantly associated with longer TSC (*p* = 0.011) and larger OSC-A (*p* = 0.014) and NOSC-A (*p* = 0.001). **Conclusions**: Preoperative use of topical ripasudil was associated with preservation of SC lumen morphology, particularly in regions lacking SC endothelium. These findings provide a theoretical basis for therapeutic strategies employing ROCK inhibitors to maintain SC morphology and function.

## 1. Introduction

Glaucoma is a major cause of irreversible blindness, affecting more than 70 million people worldwide [1,2,3]. It is estimated that approximately 5.0% of individuals aged 40 years or older are affected by glaucoma [4]. Glaucoma is characterized by optic neuropathy associated with elevated intraocular pressure (IOP), which leads to progressive retinal ganglion cell death and subsequent visual field loss [5,6,7,8,9]. The onset and progression of glaucoma are influenced by multiple factors [10,11]. Currently, the only established therapeutic approach for glaucoma is to reduce IOP, which slows disease progression and helps maintain patients’ quality of life [12,13,14].

Elevated intraocular pressure (IOP) is the main risk factor for open-angle glaucoma (OAG), including primary open-angle glaucoma (POAG) [15]. In OAG, elevated IOP is caused by decreased aqueous humor outflow from the anterior chamber (AC) into Schlemm’s canal (SC) through the trabecular meshwork/SC endothelium (SCE) complex [16,17,18]. Glaucoma treatment has advanced considerably, both surgically and pharmacologically. The Rho-associated protein kinase (ROCK) inhibitor ripasudil hydrochloride hydrate (ripasudil) emerged as a novel drug class and has been commercially available in Japan since 2014 [19,20,21,22]. It has been shown to achieve effective IOP reduction when added to a prostanoid FP receptor agonist or a β-adrenergic receptor blocker [23,24,25]. Ripasudil was the first ROCK inhibitor to be commercially marketed worldwide for the treatment of glaucoma, with its initial launch in Japan [23,24,25]. Other major classes of glaucoma medications available in Japan primarily facilitate aqueous humor drainage via the uveoscleral pathway or reduce aqueous humor production. In contrast, ripasudil increases conventional outflow through its ROCK inhibitory action [26].

Recent studies have suggested that prolonged use of glaucoma medications may result in disuse atrophy of the trabecular (conventional) outflow pathway [27]. In our previous study, we found that, in patients with primary POAG and exfoliation glaucoma (EXG), a greater number of preoperative topical medications was associated with a longer occluded segment of SC [28]. Despite advances in glaucoma pharmacotherapy, the effects of agents such as ripasudil on SC at the cellular and structural levels remain incompletely understood. Elucidating these effects could facilitate the development of improved therapies targeting the conventional outflow pathway.

This study aimed to evaluate the structural changes induced by ripasudil and their potential therapeutic value in glaucoma management. Given the pharmacological actions of ripasudil, it was hypothesized that the drug could help preserve the morphological integrity of the SC lumen. In this study, tissue specimens obtained during trabeculectomy were analyzed to compare the length and area parameters of SC morphology between eyes with and without preoperative ripasudil use.

## 2. Materials and Methods

### 2.1. Study Design and Subjects

This retrospective study was conducted in accordance with the Declaration of Helsinki and was approved by the Institutional Review Board (IRB) of Shimane University Hospital (IRB no. 20240617-1). IRB approval did not require individual written informed consent for publication; instead, the study protocol was made available at the research institution to allow participants to opt out. All specimens were obtained from a tissue library at the Japanese Red Cross Hospital [16,29]. At the Japanese Red Cross Hospital, histopathological specimens of trabecular meshwork tissue routinely excised during trabeculectomy are preserved as part of standard practice [16,29]. A total of 182 consecutive specimens from 152 patients with POAG were obtained from the library. The diagnosis of POAG was made by one author (TH) based on comprehensive ophthalmic examinations, including IOP measurement, gonioscopic evaluation, fundus examination, and visual field testing. In particular, primary angle-closure disease was carefully excluded, as were secondary glaucomas such as exfoliation glaucoma, uveitic glaucoma, and neovascular glaucoma. Sixty-two specimens that did not meet the criteria for eye drop components (described below) and 25 specimens unsuitable for morphological analysis due to tissue damage were excluded. Ultimately, 95 specimens from 95 patients were included in the final analysis.

AC gonio specimens were obtained at the time of trabeculectomy at the Japanese Red Cross Hospital Medical Center between January 1997 and June 2018. All specimens had been collected and stored with ethical approval at the Japanese Red Cross Hospital Medical Center and with written informed consent from the patients. All trabeculectomies were performed by one author (TH), and gonio specimens were collected from the upper corneoscleral limbus between the 10 and 2 o’clock positions during surgery [16,29]. Based on the criteria for eye drop components, patients were divided into two groups: those using four topical anti-glaucoma medications—FP receptor agonist, β-blocker, carbonic anhydrase inhibitor (CAI), and α_2_ agonist [ripasudil (−) group]—and those using these four components plus ripasudil [ripasudil (+) group]. Most patients were using multiple medications; therefore, the comparison was made between the four-component regimen and the four-component regimen plus ripasudil, as this classification included the largest number of patients. Patients using other medication combinations or three or fewer components were excluded.

### 2.2. Measurement of SC Morphology Parameters

The procedures for trabeculectomy, specimen preparation, and staining have been described previously [16,29]. Briefly, specimens were fixed in 2.5% formalin and 1% glutaraldehyde, divided into three to five sections, embedded in paraffin, and sectioned at 3 µm. Paraffin sections were stained with hematoxylin-eosin (HE) and immunohistochemically with anti-thrombomodulin (TBM), a marker for SCE [16,29,30]. Stained sections were photographed using an optical microscope system (OLYMPUS BX53, Tokyo, Japan; objective lens ×40, 2448 × 1920 pixels, TIFF format; a resolution of approximately 0.14 µm per pixel.). Both SC length and area parameters were measured using ImageJ software version 1.52a (National Institutes of Health, Bethesda, MD, USA) on a Windows 10 computer, as described previously [28]. The anterior and posterior edges of SC were identified in HE-stained images.

**Figure 1:**
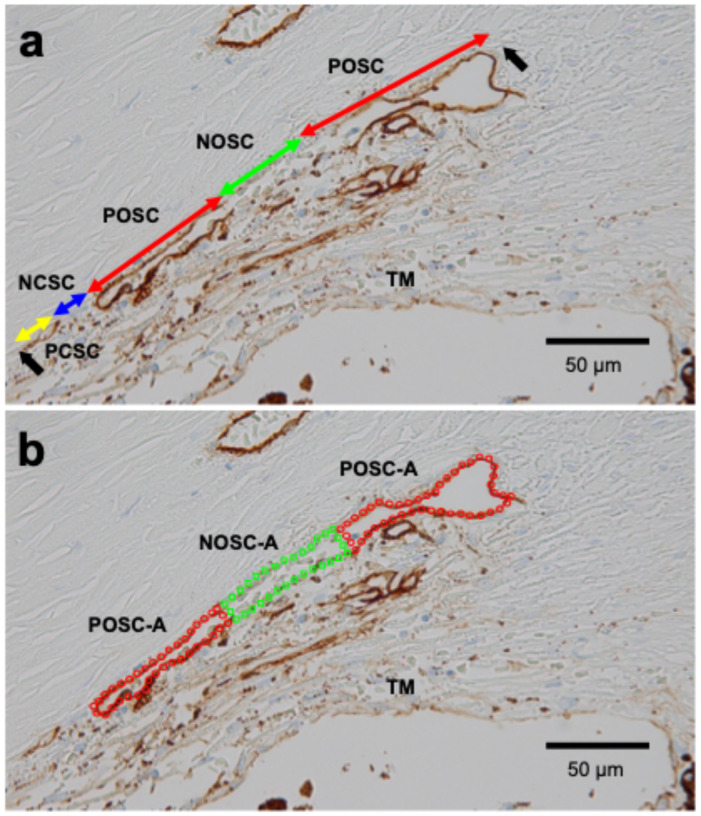
Representative measurement of SC morphology parameters in TBM-stained specimens from a patient with POAG (82-year-old female) (**a**) Red arrows indicate TBM-positive and opened SC length (POSC), the green arrow indicates TBM-negative and opened SC length (NOSC), the blue arrow indicates TBM-negative and closed SC length (NCSC), and the yellow arrow indicates TBM-positive and closed SC length (PCSC). Black arrows indicate both ends of the SC. (**b**) The area enclosed by red dots indicates TBM-positive and opened SC area (POSC-A), and the area enclosed by green dots indicates TBM-negative and opened SC area (NOSC-A).

Definitions of SC length and area parameters are shown in Table 1. Four primary length parameters were measured on TBM-stained images according to TBM staining positivity and the presence or absence of SC lumen (Figure 1a) [28]: TBM-positive with open lumen (POSC, red arrows), TBM-positive with closed lumen (PCSC, yellow arrow), TBM-negative with open lumen (NOSC, green arrow), and TBM-negative with closed lumen (NCSC, blue arrow). From these, the total SC length (POSC + PCSC + NOSC + NCSC), TBM-positive/negative SC lengths (PSC/NSC), and open/closed SC lengths (OSC/CSC) were calculated. For area parameters, the POSC area (POSC-A) was defined as TBM-positive with open lumen (red circle in Figure 1b), and the NOSC area (NOSC-A) was defined as TBM-negative with open lumen (green rectangle). OSC area (OSC-A) was calculated as POSC-A + NOSC-A. SC length parameters were measured by consensus between two examiners (AT and TH), and SC area parameters were measured by a different pair of examiners (AT and ASM). Typically, three sections were preserved for each case; measurements were taken from each section, and the mean value was used.

### 2.3. Statistical Analysis

SC morphology parameters were compared between ripasudil (+) and ripasudil (−) groups using unpaired *t*-tests for continuous variables and Fisher’s exact probability test for categorical variables. Multiple regression analysis was performed to adjust for the influence of background parameters (age, gender, preoperative IOP, use of oral CAI, and use of ripasudil), with each length and area SC morphology parameter analyzed as a dependent variable. A *p*-value of less than 0.05 was considered statistically significant. All statistical analyses were conducted using JMP Student Edition version 18.2.0 (SAS Institute Inc., Cary, NC, USA).

## 3. Results

The demographic data of the subjects are shown in Table 2. All cases in the ripasudil (−) group had been treated with four topical components (FP agonist, β-blocker, CAI, and α_2_ agonist), whereas the ripasudil (+) group received five components, including ripasudil in addition to the same four. There were no significant differences in background factors—such as age, gender, preoperative IOP, and use of oral CAI—between the two groups, except for the number of topical medications.

Univariate analysis was conducted to compare SC parameters between the two groups (Table 3). Among the length parameters, TSC was significantly longer in the ripasudil (+) group (302.5 µm) than in the ripasudil (−) group (273.0 µm, *p* = 0.023). OSC was also longer in the ripasudil (+) group (219.3 µm) compared with the ripasudil (−) group (187.4 µm), but the difference did not reach statistical significance (*p* = 0.080). Among the area parameters, OSC-A was significantly larger in the ripasudil (+) group (2689 µm^2^) than in the ripasudil (−) group (1881 µm^2^, *p* = 0.008), and NOSC-A was significantly larger in the ripasudil (+) group (554 µm^2^) compared with the ripasudil (−) group (299 µm^2^, *p* = 0.001). POSC-A did not differ significantly between the ripasudil (+) group (2001 µm^2^) and the ripasudil (−) group (1575 µm^2^, *p* = 0.096).

To account for potential confounding factors, multiple regression analysis was performed, including age, gender, preoperative IOP, use of oral CAI, and use of ripasudil as variables (Table 4). Age significantly influenced SC morphology: PSC (*p* = 0.007) and POSC (*p* = 0.005) decreased with age, whereas NSC (*p* < 0.001), CSC (*p* < 0.001), and NCSC (*p* < 0.001) increased with age. Furthermore, OSC-A (*p* = 0.045) and POSC-A (*p* = 0.007) decreased with age. Preoperative IOP was also associated with SC morphology, with higher preoperative IOP correlating with shorter PSC (*p* < 0.001), longer NSC (*p* = 0.034), and longer NCSC (*p* = 0.022). Gender and oral CAI use did not have a significant impact on any SC morphology parameter. After adjustment for these background factors, ripasudil use remained significantly associated with longer TSC (*p* = 0.011) and larger OSC-A (*p* = 0.014) and NOSC-A (*p* = 0.001).

## 4. Discussion

The present study was designed to analyze the impact of preoperative topical ripasudil use on SC morphology in patients with POAG. The results showed that the ripasudil (+) group had a significantly longer TSC and significantly larger OSC-A and NOSC-A compared with the ripasudil (−) group. Each class of glaucoma medication has a distinct IOP-lowering mechanism. β-blockers and CAI reduce aqueous humor production, FP receptor agonists increase uveoscleral outflow, and α_2_ agonists exert both effects [31,32,33]. In contrast, ROCK inhibitors lower IOP by increasing outflow through the TM–SC pathway [26,34,35]. In the present study, the two groups were comparable in glaucoma subtype and background factors other than ripasudil use (Table 1). Therefore, the distinct pharmacological action of ripasudil on the TM–SC pathway is likely to account for our findings.

In the length parameter comparison, TSC was significantly longer in the ripasudil (+) group than in the ripasudil (−) group. However, parameters related to SC opening status (OSC, CSC) or SCE presence (PSC, NSC) did not differ significantly between groups (Table 3). This prompted us to measure area parameters. OSC-A was significantly larger in the ripasudil (+) group (Table 3), and this difference remained significant after adjusting for background factors (Table 4). While OSC tended to be longer in the ripasudil (+) group, the difference did not reach significance. Since length parameters measure only the longitudinal extent of SC, they may not fully reflect the degree of canal dilation. These findings suggest that area parameters may be more sensitive than length parameters in detecting ripasudil’s effects on SC morphology. ROCK, a ubiquitous signaling mediator downstream of Rho, is activated by various bioactive factors in the AH [26,34,35]. Rho–ROCK signaling regulates essential cellular processes including adhesion, motility, proliferation, differentiation, and apoptosis [26,34,35]. Continuous reduction in conventional outflow—whether from chronic use of glaucoma medications such as FP receptor agonists [27], after successful filtration surgery [36], or from prolonged hypotony [37]—may cause disuse atrophy of the pathway. Therefore, maintenance of AH inflow into SC with ripasudil use may explain the larger OSC-A observed in the ripasudil (+) group. This aligns with previous findings showing that preoperative ripasudil use can enhance the IOP-lowering effect of microhook ab interno trabeculotomy [27].

Among area parameters, NOSC-A was notably larger in the ripasudil (+) group (Table 3 and Table 4). Previous studies have shown that negative staining for SCE markers such as TBM, CD31, and CD34 is consistent across serial sections [29] and that such areas lack SCE as confirmed by electron microscopy [30]. These results indicate that negative staining reflects loss of SCE rather than reduced marker expression. Thus, our findings suggest that ripasudil’s canal-lumen-preserving effect is more pronounced in regions lacking SCE. TM cells share contractile characteristics with smooth muscle cells, enabling them to regulate aqueous outflow [38,39,40]. The ROCK inhibitor Y-27632 induces TM cell relaxation, actin stress fiber disassembly, and focal adhesion loss, potentially lowering outflow resistance by increasing paracellular fluid flow or altering the juxtacanalicular tissue pathway [38,39,40]. Ripasudil lowers IOP by inducing retraction and rounding of TM cell bodies and disrupting actin bundles [41]. SCE is also an important target for IOP reduction [42]. Y-27632 and ripasudil increase SCE permeability, presumably through actin stress fiber loss and reduced cell stiffness/contractility [38,41]. While SC tends to be open where SCE is intact (POSC > PCSC, Table 3), it tends to be closed in regions lacking SCE (NCSC > NOSC, Table 3). Thus, in SCE-intact areas, SC may already be maximally open, limiting additional ripasudil effects, whereas in SCE-deficient areas, ripasudil’s enhancement of AH inflow into SC may be more apparent. ROCK inhibitors also exhibit antifibrotic activity, as shown in an in vivo glucocorticoid-induced ocular hypertension model [43], consistent with earlier studies [44,45,46]. Therefore, in ghost-vessel-like SC lacking SCE [47], ripasudil might directly preserve lumen structure through antifibrotic mechanisms. Our previous work suggested that SCE loss may be more pronounced in EXG than in POAG [28]. The IOP-lowering effect of ripasudil may be comparable or even stronger in secondary glaucoma than in POAG [48,49], potentially due to differences in SCE damage between subtypes.

In multivariate analysis, SC morphology showed age-related changes: PSC, POSC, OSC-A, and POSC-A decreased with age, whereas NSC, CSC, and NCSC increased (Table 4). These trends are consistent with previous findings showing reduced SC diameter and area, and increased TM thickness with age [50]. Higher preoperative IOP was associated with shorter PSC and longer NCSC (Table 4), in agreement with known clinical and histological relationships. Collectively, these results support the validity of our analytical approach for evaluating clinical specimens.

Several limitations should be noted. Only a small portion of SC tissue was examined, which may not fully represent SC/SCE changes in the whole eye. The lack of visual field, fundus, and gonioscopic findings is another limitation, as these background factors were unavailable from the existing tissue library we used. A new tissue library with detailed clinical data is currently being developed to enable future studies correlating histological findings with clinical parameters. Another consideration is that the potential influence of medications other than ripasudil on SC/SCE morphology cannot be entirely excluded. As recommended by the Japan Glaucoma Society Guidelines, FP receptor agonists and β-blockers are typically used as first-line agents, with CAI, α_2_ agonists, and ROCK inhibitors as second-line options [51]. Trabeculectomy, while highly effective in lowering IOP, carries risks of complications [52] and is usually chosen after maximal tolerated medical therapy. Indeed, most cases in our tissue library had received multiple topical medications in various combinations. To maximize sample size for evaluating ripasudil’s effects, we focused on eyes receiving four components with or without ripasudil. Although a comparison between ripasudil monotherapy and untreated eyes would be ideal, it is impractical in clinical settings. Although no significant difference was observed between the groups, the 95% confidence interval of preoperative IOP was wider in the ripasudil (+) group than in the ripasudil (−) group. Because patients in the ripasudil group were receiving five classes of antiglaucoma medications, this group may have included more severe cases with greater elevations in IOP. In general, morphological changes in SC/SCE are expected to be more pronounced in more severe cases. Therefore, the present comparison may have underestimated the effects of ripasudil on SC/SCE. The absence of group differences in background factors and the use of multivariate adjustment strengthen the validity of our design for assessing ripasudil’s effects. The absence of a significant difference in POSC-A may be largely attributable to low statistical power, suggesting that the relatively small sample size is another limitation of the study. SC/SCE morphology differs among glaucoma subtypes [28]. Although the present study focused exclusively on POAG, we are currently investigating the effects of ripasudil in other glaucoma subtypes. In addition, information on treatment duration and sequence was unavailable. Since all included eyes had insufficient IOP control despite maximal therapy, our findings may underestimate ripasudil’s true effects on SC/SCE morphology.

## 5. Conclusions

In conclusion, this study demonstrated that topical ROCK inhibitor use may preserve SC lumen structure in human samples. These findings provide a theoretical basis for therapeutic strategies employing ROCK inhibitors to maintain SC morphology and function.

## Figures and Tables

**Table 1:** Definition of parameters.

Abbreviation	Parameter Name	Definition
TSC	Total SC length	POSC + PCSC + NOSC + NCSC
PSC	TBM positive SC length	POSC + PCSC
NSC	TBM negative SC length	NOSC + NCSC
OSC	Opened SC length	POSC + NOSC
CSC	Closed SC length	PCSC + NCSC
POSC	TBM positive and opened SC length	Length of TBM positive and opened SC on TBM staining
PCSC	TBM positive and closed SC length	Length of TBM positive and closed SC on TBM staining
NOSC	TBM negative and opened SC length	Length of TBM negative and opened SC on TBM staining
NCSC	TBM negative and closed SC length	Length of TBM negative and closed SC on TBM staining
OSC-A	Opened SC area	POSC-A + NOSC-A
POSC-A	TBM positive and opened SC area	Area of TBM positive and opened SC on TBM staining
NOSC-A	TBM negative and opened SC area	Area of TBM negative and opened SC on TBM staining

SC, Schlemm’s canal; TBM, thrombomodulin immuno-staining.

**Table 2:** Multivariable linear regression analysis of ACCI and time-domain HRV parameters.

Parameters	Ripasudil (−) (*n* = 68)	Ripasudil (+) (*n* = 27)	*p*-Value
**Number of topical medications**			
	4	5	-
**Age (years)**			
Mean ± SD	64.9 ± 11.7	60.2 ± 11.7	0.079
95% CI	62.1, 67.8	55.6, 64.8	
**Gender**			
Male, *n* (%)	36 (53)	16 (59)	0.651
Female, *n* (%)	32 (47)	11 (41)	
**Preoperative IOP (mmHg)**			
Mean ± SD	23.0 ± 4.7	24.5 ± 10.2	0.311
95% CI	21.8, 24.1	20.5, 28.5	
**Oral CAI**			
Yes, *n* (%)	16 (24)	7 (26)	0.796
No, *n* (%)	52 (76)	20 (74)	

Continuous variables are compared by *t*-test, and categorial variables are compared by Fisher’s exact probability test. SD, standard deviation; CI, confidence interval; IOP, intraocular pressure; CAI, carbonic anhydrase inhibitor.

**Table 3:** Univariate analysis for comparison of measured SC morphological parameters between ripasudil (−) and (+) groups.

Paramater		Ripasudil (−)	Ripasudil (+)	*p*-Value
TSC (µm)	Mean ± SD	273.0 ± 59.0	302.5 ± 46.7	0.023 *
	95% CI	258.8, 287.3	284.0, 320.9	
PSC (µm)	Mean ± SD	170.4 ± 68.8	181.1 ± 66.9	0.494
	95% CI	153.8, 187.1	154.6, 207.6	
NSC (µm)	Mean ± SD	102.6 ± 66.5	121.4± 71.9	0.229
	95% CI	86.5, 118.7	92.9, 149.8	
OSC (µm)	Mean ± SD	187.4 ± 81.0	219.3 ± 74.9	0.080
	95% CI	167.8, 207.0	189.7, 249.0	
CSC (µm)	Mean ± SD	85.6 ± 68.9	83.1 ± 76.9	0.877
	95% CI	69.0, 102.3	52.7, 113.5	
POSC (µm)	Mean ± SD	147.0 ± 71.3	162.2 ± 62.9	0.336
	95% CI	129.7, 162.3	137.3, 187.1	
PCSC (µm)	Mean ± SD	23.4 ± 32.4	18.9 ± 24.5	0.513
	95% CI	15.6, 31.3	9.2, 28.6	
NOSC (µm)	Mean ± SD	40.4 ± 49.0	57.1 ± 50.2	0.139
	95% CI	28.5, 52.2	37.3, 77.0	
NCSC (µm)	Mean ± SD	62.2 ± 54.4	64.2 ± 75.4	0.884
	95% CI	49.1, 75.4	34.4, 94.1	
OSC-A (µm^2^)	Mean ± SD	1881 ± 1136	2689 ± 1681	0.008 **
	95% CI	1606, 2156	2023, 3354	
POSC-A (µm^2^)	Mean ± SD	1575 ±1046	2001 ± 1273	0.096
	95% CI	1322, 1828	1497, 2504	
NOSC-A (µm^2^)	Mean ± SD	305 ± 386	716 ± 774	0.001 **
	95% CI	212, 399	410, 1022	

*p*-values are calculated by *t*-test. The * and ** indicates significance levels of 5% (*p* < 0.05) and 1% (*p* < 0.01), respectively. SD, Standard deviation; CI, Confidence interval; SC, Schlemm’s canal; TSC, total SC length; PSC, positive SC length; NSC, negative SC length; OSC, opened SC length; CSC, closed SC length; POSC, positive and opened SC length; PCSC, positive and closed SC length; NOSC, negative and opened SC length; NCSC, negative and closed SC length; OSC-A, opened SC area; POSC-A, positive and opened SC area; NOSC-A, negative and opened SC area.

**Table 4:** Multivariate analysis for association between SC morphological parameters and various background parameters.

Paramater		Age (/Year)	Gender (Female/Male)	Pre-Operative IOP (/mmHg)	Oral CAI (Yes/No)	Ripasudil (Yes/No)
TSC (µm)	r	0.9	−3.3	−0.7	7.2	17.0
	95% CI	−0.1, 1.8	−5.3, 8.7	−2.7, 1.2	−8.0, 22.3	4.0, 29.8
	*p*-value	0.089	0.584	0.472	0.350	0.011 *
PSC (µm)	r	−1.6	−7.3	−3.0	−0.15	3.5
	95% CI	−2.7, −0.4	20.9, 6.4	−5.2, −0.7	−17.4, 17.1	−11.2, 18.2
	*p*-value	0.007 **	0.293	<0.001 **	0.986	0.639
NSC (µm)	r	2.4	4.0	2.3	7.3	13.4
	95% CI	1.4, 3.5	−8.9, 16.8	0.2, 4.4	−8.9, 23.5	−0.4, 27.2
	*p*-value	<0.001 **	0.541	0.034 *	0.372	0.057
OSC (µm)	r	−1.3	−8.3	−2.1	−8.3	14.2
	95% CI	−2.7, 0.1	−24.8, 8.2	−4.8, 0.6	−29.2, 12.6	−3.6, 32.0
	*p*-value	0.059	0.322	0.117	0.430	0.116
CSC (µm)	r	2.2	5.0	1.4	15.5	2.7
	95% CI	1.0, 3.3	−8.9, 18.8	−0.8, 3.7	−2.0, 33.0	−12.2, 17.6
	*p*-value	<0.001 **	0.480	0.212	0.082	0.721
POSC (µm)	r	−1.7	−11.0	−2.3	−3.9	4.8
	95% CI	−2.8, −0.5	−24.9, 2.9	−4.5, 0.0	−21.5, 13.7	−10.1, 19.8
	*p*-value	0.005 **	0.120	0.051	0.662	0.523
PCSC (µm)	r	0.1	3.7	−0.7	3.7	−1.3
	95% CI	−0.4, 0.6	−2.8, 10.2	−1.8, 0.3	−4.5, 12.0	−8.4, 5.7
	*p*-value	0.721	0.264	0.181	0.371	0.706
NOSC (µm)	r	0.4	2.7	0.1	−4.4	9.4
	95% CI	−0.5, 1.2	−8.1, 13.5	−1.6, 1.9	−18.1, 9.2	−2.2, 21.0
	*p*-value	0.431	0.620	0.899	0.519	0.112
NCSC (µm)	r	2.1	1.3	2.2	11.8	4.0
	95% CI	1.1, 3.0	−10.0, 12.5	0.3, 4.0	−2.5, 26.0	−8.1, 16.2
	*p*-value	<0.001 **	0.825	0.022 *	0.105	0.512
OSC-A (µm^2^)	r	−22.8	−220.1	−37.1	−106.0	367.5
	95% CI	−45.1, −0.5	−490.6, 50.4	−81.3, 7.0	−447.9, 235.9	76.4, 658.6
	*p*-value	0.045 *	0.109	0.098	0.539	0.014 *
POSC-A (µm^2^)	r	−25.5	−194.4	−34.8	−91.5	169.5
	95% CI	−43.9, −7.0	−418.5, 29.7	−71.4, 1.8	−374.7, 191.8	−71.7, 410.7
	*p*-value	0.007 **	0.088	0.062	0.523	0.166
NOSC-A (µm^2^)	r	3.5	−14.1	0.1	−9.3	212.6
	95% CI	−6.0, 13.0	−129.1, 100.8	−18.7, 18.9	−154.6, 136.0	88.9, 336.3
	*p*-value	0.465	0.807	0.991	0.899	0.001 **

*p* and r (unstandardized regression coefficient) values are calculated by multiple regression analysis. The * and ** indicate significance levels of 5% (*p* < 0.05) and 1% (*p* < 0.01), respectively. CI, Confidence interval; SC, Schlemm’s canal; TSC, total SC length; PSC, positive SC length; NSC, negative SC length; OSC, opened SC length; CSC, closed SC length; POSC, positive and opened length; PCSC, positive and closed SC length; NOSC, negative and opened SC length; NCSC, negative and closed SC length; OSC-A, opened SC area; POSC-A, positive and opened SC area; NOSC-A, negative and opened SC area; IOP, intraocular pressure; CAI, carbonic anhydrase inhibitor.

## Data Availability

Data is fully available upon reasonable request to corresponding authors.

## Abbreviations

The following abbreviations are used in this manuscript:

AC	anterior chamber
CAI	carbonic anhydrase inhibitor
CI	confidence interval
EXG	exfoliation glaucoma
HE	hematoxylin-eosin
IOP	intraocular pressure
IRB	Institutional Review Board
OAG	open-angle glaucoma
POAG	primary open-angle glaucoma
ROCK	Rho-associated protein kinase
SC	Schlemm’s canal
SCE	Schlemm’s canal endothelium
SD	standard deviation
TBM	thrombomodulin
TM	trabecular meshwork
